# Anatomy and Physiology

**Published:** 1840-01-01

**Authors:** 


### ANATOMY AND PHYSIOLOGY.

1. The Cyclopædia of Anatomy and Physiology. Edited by *Robert Todd*, M.D. F.R.S. &c. &c. Parts XV. XVI. XVII. and XVIII.

We have hitherto omitted noticing the latter Parts of this excellent work. This has been owing to the press of other matter, for the work itself has maintained its character and continued to do credit to its writers and its editor.

We shall select from among the articles contained in the Parts before us, two or three of a physiological, and generally interesting character. Our senior readers will not, perhaps, be displeased at occasionally having the opportunity afforded them of bringing up their knowledge of physiology to the actual level of the day.

The first subject to which we shall allude is hibernation. It has been treated very happily by Dr. Marshall Hall, who has devoted, as our readers must be well aware, considerable pains and his usual talents to its investigation.

### I. On Hibernation.

Dr. Hall observes:

"It has not hitherto been distinctly ascertained to what extent the state of hibernation prevails in the animal kingdom; the bat, the hedgehog, and the dormouse, are the genera which present us with the most marked examples of this singular physiological condition in this country; to these the elegant authoress of 'Sketches of Natural History' has added the water-rat and the wood-mouse, observing of the former—

"And when cold winter comes and the water-plants die,
And his little brooks yield him no longer supply,
Down into his burrow he cozily creeps,
And quietly through the long winter-time sleeps."

The following quotation will be found to afford the key to Dr. Hall's views on the nature of hibernation. It is long but indispensable.

"There is in my opinion, an ultimate *law* of animal existence, which seems to regulate the different forms in which the different classes of animals present themselves. The quantity of respiration is inversely as the degree of irritability of the muscular fibre, the former being marked by the quantity of oxygen consumed in a given time, ascertained by the pneumatometer, the latter by the force of galvanism necessary to demonstrate its existence. The bird tribes have a high respiration and a low irritability; the reptiles have a high degree of irritability and little respiration. This law obtains not only in the different tribes of animals, but also in the different stages or states of the same individual, the *structural* changes from one stage to another being always a change from a lower to a higher respiration, and from a higher to a lower degree of irritability, and the change of state, a change in the opposite direction: thus the changes from the egg to the bird, from the tadpole to the batrachian form, from the larva to the chrysalis and the insect condition, are changes in which, whilst a due ratio is constantly maintained, the quantity of respiration is augmented and the degree of irritability diminished; on the other hand, the *physiological* changes in the degree of activity in animals, during sleep, for example, but especially in that remarkable change which is the subject of this article, the respiration is diminished whilst the degree of irritability is, *pari passu,* augmented.

On what this susceptibility of change depends, and especially on what the power of taking on an augmented irritability depends, is at present unknown. But I think I may affirm that it is upon this power that the capability of passing into the state of hibernation reposes. I suppose that all animals have the faculty of sleeping; during sleep the respiration is slightly diminished, the irritability probably proportionately augmented—probably one ultimate object of this state of repose; but the phenomenon has its appointed limit which it cannot pass. In certain animals, that limit is not so confined,—the quantity of respiration is still further diminished, the degree of irritability still further augmented, and the deeper sleep, or lethargy, of hibernation takes place.

During this lethargy, the law of the inverse ratio of the respiration and of the irritability still prevails, and the animal merely puts on a reptile state in these respects. Were the respiration to be diminished without the appointed augmentation of the irritability, the heart would cease to be stimulated, and the animal would die, as in the cases of torpor and slow asphyxia; were the respiration augmented without the proportionate diminution of the irritability, the heart would be over-stimulated, and death would alike ensue, as in the case of a hibernating animal too suddenly roused from its lethargy, and as (probably) in the case of an animal placed in pure oxygen gas.

The difference between the hibernating and all other animals then is, an ultimate faculty of assuming an augmented degree of irritability of the muscular fibre—a power possessed by all animals within certain limits, but by the hibernating animal beyond the usual limit." 765.

This is the dominant idea of the article. It has been generally believed that hibernation depends on the animal's not being able to generate heat sufficiently to resist a low temperature. Dr. Hall lays it to the faculty of assuming an augmented degree of irritability of muscular fibre. It must be owned that neither theory explains the phenomenon satisfactorily. Milne Edwards the advocate of the former, remarks:—" Nous voyons que les chauves-souris produisent habituellement moins de chaleur que les animaux à sang chaud, et que c'est principalement à cette cause qu'il faut attribuer l'abaissement de leur température pendant la saison froide. En comparant cette expérience sur la chauve-souris adulte avec celles que nous avons faites sur les jeunes animaux à sang chaud, on y aperçoit un rapport remarquable; ils ne produisent pas assez de chaleur pour soutenir une température élevée, lorsque l'air est à un degré voisin de zéro. Mais il y a cette difference, que c'est un état passager chez les jeunes animaux à sang chaud, et qu'il est permanent chez les chauves-souris.

" Il est évident que les autres mammifères hibernans doivent participer plus ou moins de cette manière d'être. Les faits que j'ai exposés suffisent pour nous faire considérer ce groupe d'animaux sous le point de vue suivant; qu'au printemps et en été, dans leur état d'activité et de veille, lorsque leur température est assez élevée pour ne pas différer essentiellement de celle qui caractérise les animaux à sang chaud, ils n'ont pas la faculté de produire autant de chaleur; et tout en admettant que d'autres causes peuvent influer sur leur refroidissement pendant leur hibernation, il faut cependant l'attribuer en grande partie à cette particularité de leur constitution." But as Dr. Hall observes this explanation would account for torpor, a pathological and a fatal state, not for hibernation, a natural and a harmless one. Dr. Hall's own explanation does little more than put the fact of hibernation in scientific terms—when analysed it resolves itself into the expression, *that* certain animals have the power of hibernating. To say that the irritability of the muscular fibre is increased, is to say little else than all were aware of, for as the heart was known to beat while the respiration was imperfect, and the changes in the blood incomplete, it followed of course that the heart contracted under the influence of a feeble stimulus, or, in other words, that its irritability was augmented.* The *how* is left involved in the same obscurity as heretofore; indeed, supposing Milne Edwards' explanation unobjectionable, it would be more satisfactory as an explanation than that of Dr. Hall.

This gentleman not only compares hibernation with sleep, but he even goes so far as to believe that it is nothing but profound sleep. He says:—Hibernation appears more extraordinary only because less familiar than sleep. Most animals are, in fact, naturally awake and asleep every revolving day, some being diurnal, others nocturnal. But in summer the bat actually hibernates, loses its respiration, and with its respiration its temperature, acquires vastly augmented irritability, and presents the other phenomena of *complete* hibernation, regularly and periodically every twenty-four hours; and the hedgehog and the dormouse present similar phenomena, only after other intervals.

This would seem to shew that Dr. Hall looks on the law of periodicity, whatever that may be, as being essentially the law of hibernation. He holds the influence of diminished temperature cheap. He goes on to state:—

"Sleep then is the first stage of hibernation. The faculty of passing into the second is identical with that of assuming a greatly augmented irritability of the muscular fibre. Such are the results of my long attention to this interesting physiological question. Much error has arisen from viewing hibernation as a simple effect of cold. The influence of cold in inducing hibernation is merely its well-known influence in inducing sleep, concurring with the other causes of this condition. The direct effect of cold on the animal frame is, as I shall shortly have occasion to state particularly, totally different from hibernation. Hibernation is a physiological condition; the direct effect of cold, or *torpor*, is, on the contrary, a pathological and generally a fatal one.

The term hibernation has usually been applied to designate what its etymology implies, the condition in which certain animals pass the *winter* season. An error is, as I have already stated, involved in this view of the subject; for the condition termed hibernation is not confined to the winter season. Cuvier observes, in speaking of the Tenrecs, 'ce sont des animaux nocturnes qui passent trois mois de l'anné en lethargie, quoique habitants de la zône torride. Burguière assure même que c'est pendant les grandes chaleurs qu'ils dorment.' Hence the term *Sommerschlaf* employed in Germany. It is plain too, from this circumstance, that the state of hibernation is not necessarily connected with a low degree of external temperature, and we are surprised to find this celebrated naturalist, whom I have just quoted, observing, 'la seule condition de la lethargie est le froid et l'absence des causes irritantes.'

I must repeat that hibernation is, in every respect, but the parallel of ordinary sleep, varying only in force and duration. It is equally modified by co-operating

---

* It must, on consideration, be obvious to Dr. Hall himself, that he has enunciated no new law. We were all aware that the reptilia and amphibia whose blood is but semi-aerated are distinguished for irritability of muscular fibre.

or opposing causes; and it is equally manifested in its peculiar effects, only varying in degree and intensity." 766.

Dr. Hall goes on to remark that, to understand hibernation, we must look at it as it affects every function; and he deems it necessary to ascertain first what sleep, the initiative of hibernation, is. Sleep affects the cerebral system only—the true spinal, or excito-motory retains all its energies. But, he remarks, in the state of activity, the cerebral system exerts a peculiar and continual influence over the true spinal, which ceases during sleep. In this manner the functions of the latter appear impaired; the respiration especially, and with the respiration the circulation, with which it always maintains a certain relation, becomes slower, irregular, and suspended at intervals. These phenomena observable in ordinary sleep are still more remarkable in the deep sleep or lethargy of hibernation or diurnation.

*Of the Sleep of Hibernating Animals.*—Dr. Hall states, as the result of his own observations, *that*, in the sleep of the hibernating animal, the respiration is more or less impaired: if the animal be placed in circumstances which best admit of observation, the acts of respiration will be found to have greatly diminished; if it be placed in the pneumatometer, little alteration is induced in the bulk of the air; if its temperature be taken by the thermometer, it will be found to be many degrees lower than that of the animal in its active state; if it be deprived of atmospheric air, it is not immediately incommoded or injured.

"The bat, which is a crepuscular or nocturnal feeder, regularly passes from its state of activity to one which may be designated *diurnation*. The respiration and the temperature fail; the necessity for respiration is greatly lessened.

During the summer of 1831, I carefully observed a bat in this condition. If it were quite quiet, its respiration became very imperfect; its temperature was but a few degrees above that of the atmosphere; being placed under water, it remained during eleven minutes uninjured, and on being removed became lively and continued well.

I have more recently watched the habits of two hedgehogs, in a temperature varying from 45° to 50°. These animals alternately awake, take food, and fall asleep. One of them is frequently awake, whilst the other is dormant, and goes to sleep at a time that the other awakes, but without regularity. When awake, the temperature of each, taken by pressing the bulb of a thermometer upon the stomach, is about 95°; when dormant, it is 45°; that of the atmosphere being 42° or 43°. The duration of this sleep is from two to three days, according to the temperature of the atmosphere. On the 4th of February, 1832, the temperature of the atmosphere being 50°, both the hedgehogs were dormant,—the temperature of one was 51°, and that of the other 52°; on the succeeding day, the temperature of the atmosphere had fallen one degree, the temperature of one of the hedgehogs was 49°, whilst that of the other, which had become lively, had risen to 87°; on the succeeding day, the first had become somewhat lively, and its temperature had risen to 60°, that of the other being 85°, and that of the atmosphere 47°.

I have observed precisely the same alternations in the dormouse; except that this animal awakes daily in moderate temperatures, takes its food, and re-passes into a state of sleep, in which the respiration is greatly impeded, and the temperature little higher than that of the atmosphere.

On the day on which the observations were made on the hedgehogs, the atmosphere being 49°, that of two dormice was 52°; on the succeeding day, the external temperature being 47°, that is, lower by two degrees, the temperature of one of these dormice was 92°, and that of the other 94°; and only three hours afterwards, the temperatures were 60° and 70° respectively, with a slight appearance of lethargy.

The hedgehog and the dormouse appear, in fact, to awake from the call of hunger, then to eat, and then again to become dormant, in temperatures which may be termed moderate. The bat, which could not find food if it did awake, does not undergo these periodical changes, except in the summer season. It appears to me, from the most careful observation, that there is every degree between the ordinary sleep of these animals and the most profound hibernation.

It is quite obvious, from these observations, that the ordinary sleep of hibernating animals differs from that of others, by inducing a more impaired state of the respiration and of the evolution of heat, with an augmented power of bearing the abstraction of the atmospheric air. This sleep probably passes into true hibernation, as the blood which circulates through the brain becomes more and more venous, from the diminution of the respiration, and as the muscular fibre of the heart acquires increased irritability." 767.

Dr. Hall continues :—

It is absolutely necessary, in comparing the powers of hibernating and other animals, of evolving heat, accurately to observe whether there be any tendency to sleep. Mr. Hunter's and M. Edwards's experiments are deficient for want of this attention. Mr. Hunter, comparing the common mouse and the dormouse, exposed to a very low temperature, observes, that the temperature of the former was " diminished 16° at the diaphragm, and 18° in the pelvis; while in the dormouse it gained five degrees, but lost on a repetition." The explanation of these facts is afforded by the observation, that when the dormouse increased in temperature it was " very lively," but that on the " repetition" it had become " less lively ;" the mouse was probably in a state of languor from apprehension or for want of food.

M. Edwards omits to mention whether the hibernating animals, in his experiments, were disposed to be lively or dormant, or whether they had recently recovered from the dormant state. He does not even mention whether the experiments on the bat were performed in the evening, its period of activity, or in the morning or day, its period of lethargy or diurnation. Without a particular attention to these points, no correct result could be obtained. The hibernating animal, in a state of vigour and activity, is a totally different being from the same animal disposed to become dormant.

In order to perform this experiment in a satisfactory manner, the bat, for example, should be employed in the evening, when it has naturally awoke from its deep day-slumber, the hedgehog when it has awoke spontaneously to take food; otherwise the disposition to sleep may explain the loss of temperature.

*Of Perfect Hibernation.*—Dr. Hall offers as the abbreviated result of a series of observations and experiments made in the course of the years 1831-2, *that*, there is *one* special cause of hibernation,—that law imposed by the Creator, according to which *all* animals become affected with sleep at some period of each revolving day, and the hibernating animal at some period of the revolving year. We have thus presented to us the phenomena of diurnal and nocturnal animals, and the winter-sleep and the summer-sleep of hibernating animals.

Exposure to cold, not too severe, disposes to hibernation, as it disposes to ordinary sleep. Severe cold, on the contrary, first rouses the hibernating animal from its lethargy, and then plunges this and all animals into a state of fatal torpor.

The absence of every kind of stimulus or excitant, and a somewhat confined atmosphere,* also conduce to hibernation.

Every excitement, on the contrary, that of hunger, that of the sexes probably, tend to disturb this peculiar lethargy. It is in this manner that we explain the periodicity of sleep and hibernation, though there is probably also some hidden influence of the seasons, of the day or of the year, influences which have been traced by Dr. Prout and by M. Edwards in regard to the quantity of respiration.

Dr. Hall proceeds to examine the influence of hibernation on the several functions.

A. *Sanguification.*—" There is much difference," says our author, " in the powers of digestion, and in the fact of omitting to take food, in the hibernation of different animals. The bat, being insectivorous, would awake in vain; no food could be found: the hedgehog might obtain snails or worms, if the ground were not very hard from frost: the dormouse would find less difficulty in meeting with grain and fruits. We accordingly observe a remarkable difference in the habits of awaking from their lethargy or hibernation, in these different animals. I have observed no disposition to awake at all in the bat, except from external warmth or excitement. If the temperature be about 40° or 45°, the hedgehog, on the other hand, awakes, after various intervals of two, three, or four days passed in lethargy, to take food; and again returns to its state of hibernation. The dormouse, under similar circumstances, awakes daily.

Proportionate to the disposition to awake and take food, is the state of the functions of the stomach, bowels and kidneys. The dormouse and the hedgehog pass the fæces and urine in abundance during their intervals of activity. The bat is scarcely observed to have any excretions during its continued lethargy.

In the dormouse and the hedgehog, the sense of hunger appears to rouse the animal from its hibernation, whilst the food taken conduces to a return of the state of lethargy. It has already been observed, that there are alternations between activity and lethargy in this animal, with the taking of food, in temperatures about 40° or 45°. Nevertheless, abstinence doubtless conduces to hibernation, by rendering the system more susceptible of the influence of cold, in inducing sleep and the loss of temperature. The hedgehog, which awakes from its hibernation, and does not eat, returns to its lethargy sooner than the one which is allowed food." 769.

Dr. Hall observes that the *respiration* is nearly suspended in hibernation, which is proved, 1st, by the absence of all detectable respiratory acts; 2dly, by the almost entire absence of any change in the air of the pneumatometer; 3dly, by the subsidence of the temperature to that of the atmosphere; and 4thly, by the capability of supporting, for a great length of time, the entire privation of air.

1. "I have adopted various methods to ascertain the entire absence of the acts

---

* M. de Saissy observes, " la marmotte, que j'ai engourdie par deux fois différentes, ne l'a été, je crois, que parceque je me suis avisé, quand la respiration a été bien affaiblie, de boucher le trou du couvercle. Ce n'a été que de cette manière que je suis parvenu à l'engourdir; car toutes les tentatives que j'avais faites avant ont été vaines."

of respiration. I placed bats in small boxes, divided by a partition of silk riband, the cover of which consisted of glass, and in the side of which a small hole was made to admit of placing a long light rod or feather under the animal's stomach. The least respiratory movement caused the extremity of this rod to pass through a considerable space, so that it became perfectly apparent.

Over the hibernating hedgehog I placed a similar rod, fixing one extremity near the animal, and leaving the other to move freely over an index. During hibernation not the slightest movements of these rods could be observed, although they were diligently watched. But the least touch, the slightest shake immediately caused the bat to commence the alternate acts of respiration, whilst it invariably produced the singular effect of a deep and sonorous inspiration in the hedgehog. It is only necessary to touch the latter animal to ascertain whether it be in a state of hibernation or not: in the former case there is this deep sonorous inspiration; in the latter, the animal merely moves and coils itself up a little more closely than before. After the deep inspiration, there are a few feeble respirations, and then total quiescence. The bat makes similar respirations without the deep inspiration, and then relapses into suspended respiration.

2. As the acts of respiration are nearly suspended during hibernation, so are the changes induced in the atmospheric air.

On January the 28th, the temperature of the atmosphere being 42°, I placed a bat in the most perfect state of hibernation and undisturbed quiet, in the pneumatometer, during the whole night, a space of ten hours, from 1h. 30m. to 11h. 30m. There was no perceptible absorption of gas.

Having roused the animal a little, I replaced it in the pneumatometer, and continued to disturb it from time to time, by moving the apparatus. It continued inactive, and between the hours of 1h. 20m. and 4h. there was the absorption of one cubic inch only of gas.

Being much roused at four o'clock, and replaced in the pneumatometer, the bat now continued moving about incessantly; in one hour, five cubic inches of gas had disappeared. It was then removed. A further absorption took place of ·8 of a cubic inch of gas.

Thus the same little animal, which, in a state of hibernation, passed ten hours without respiration, absorbed or converted into carbonic acid, 5.8 cubic inches of oxygen gas in one hour when in a state of activity. In an intermediate condition, it removed one cubic inch of oxygen in two hours and forty minutes." 769.

Dr. Hall twice repeated the experiment with substantially similar results. For, 3·4 cubic inches of oxygen gas disappeared in sixty hours, from the respiration of a bat in the state of lethargy; while, during the state of activity of the animal, an equal quantity of the same gas disappeared in less than half that number of minutes.

"It may be a question whether the slight quantity of respiration I have mentioned be cutaneous. The absence of the acts of respiration would lead us to this opinion. But it may be observed, that these acts have not been watched, and can scarcely be watched continuously enough, to determine the question of their entire absence. Some contrivance to ascertain whether the rod has moved along the index during the absence of the observer would resolve every doubt upon this interesting point. And I think it right to remark, that after the apparent total cessation of respiration, as observed by the means which have just been described, there is probably still a slight diaphragmatic breathing. I am led to this conclusion, by having observed a slight movement of the flank in a favourable light, unattended by any motion of the thorax or epigastrium." 769.

4. Dr. Hall insists on the necessity of great precautions in ascertaining the comparative temperature of the animal and of the atmosphere. He subjoins a table which gives the result of observations made during many days, in very varying temperatures. From this, he says, it is obvious that the temperature of the hibernating animal accurately follows that of the atmosphere. When the changes of temperature in the latter are slight, the two thermometers denote the same temperature. If these changes are greater and more rapid, the temperature of the animal is a little lower or higher, according as the external temperature rises or falls; a little time being obviously required for the animal to attain that temperature.

It is only necessary to add to these observations, that the internal temperature is about three degrees higher than that of the epigastrium. In two bats, the external temperature of each of which was 36°, a fine thermometer, with an extremely minute cylindrical bulb, passed gently into the stomach, rose to 39°.

4. It is not astonishing that the lethargic animal is enabled to bear the total abstraction of atmospheric air or oxygen gas, for a considerable period of time. Dr. Hall remarks that:—

Spallanzani placed a marmot in carbonic acid gas, and makes the following report of the experiment in a letter to Senebier: "Vous vous ressouviendrez de ma marmotte qui fut si fortement léthargique dans l'hiver sévère de 1795; je la tins alors pendant quatre heures dans les gaz acide carbonique, le thermomètre marquant—12°, elle continua de vivre dans ce gaz qui est le plus mortel de tous, comme je vous le disais: au moine un rat et un oiseau que j'y plaçai avec elle y périrent à l'instant même. Il-parait donc que sa respiration fut suspendu pendant tout ce tems-là. Je soumis à la même experience des chauve-souris semblablement léthargiques et le résultat fut le même."

A bat which was lethargic in an atmosphere of 36° was immersed in water of 41°. It moved about a little, and expelled bubbles of air from its lungs. It was kept in the water during sixteen minutes, and then removed. It appeared to be uninjured by the experiment.

A hedgehog which had been so lethargic in an atmosphere of 40° as not to awake for food during several days, was immersed in water of 42°. It moved about and expelled air from its lungs. It was retained under the water during 22½ minutes. It was then removed. It appeared uninjured.

It seems probable that the motions observed in these animals were excited through the medium of the cutaneous nerves.

The power of supporting the abstraction of oxygen gas, or atmospheric air, belongs solely to the hibernating state, and is no property of the hibernating animal in its state of activity. Dr. Hall immersed an active hedgehog in water. It expired in three minutes, the period in which immersion proves fatal to the other mammalia.

5. "The *circulation* is reduced to an extreme degree of slowness, according to a law well-known, but hitherto, I believe, unexplained, according to which the respiration and the circulation are always proportionate to each other.

The wing of the bat affords an admirable opportunity of observing the condition of the circulation during hibernation. But it requires peculiar management. If the animal be taken from its cage, and the wing extended under the microscope, it is roused by the operation, and its respiratory and other movements are so excited, that all accurate observation of the condition of the circulation in the minute vessels is completely frustrated. Still greater caution is required in this case than even in the observation of the respiration and temperature.

After some fruitless trials, I at length succeeded perfectly in obtaining a view of the minute circulation undisturbed. Having placed the animal in its state of hibernation, in a little box of mahogany, I gently drew out its wing through a crevice made in the side of the box; I fixed the tip of the extended wing between portions of cork; I then attached the box and the cork to a piece of plate-glass; and lastly, I left the animal in this situation, in a cold atmosphere, to resume its lethargy.

I could now quietly convey the animal ready prepared, and place it in the field of the microscope without disturbing its slumbers, and observe the condition of the circulation.

In this manner I have ascertained that, although the respiration be suspended, the circulation continues uninterruptedly. It is slow in the minute arteries and veins; the beat of the heart is regular, and generally about twenty-eight times in the minute.

We might be disposed to view the condition of the circulation in the state of hibernation as being reptile or analogous to that of the batrachian tribes. But when we reflect that the respiration is nearly, if not totally, suspended, and that the blood is venous, we must view the condition of the circulation as in a lower condition still, and, as it were, sub-reptile. It may, indeed, be rather compared to that state of the circulation which is observed in the frog from which the brain and spinal marrow have been removed by minute portions at distant intervals.

In fact, in the midst of a suspended respiration, and an impaired condition of some other functions, one vital property is augmented. This is the irritability, and especially the irritability of the left side of the heart. The left side of the heart, which is, in the hibernating animal, in its state of activity, as in all the other mammalia, only arterio-contractile, becomes veno-contractile.

This phenomenon is one of the most remarkable presented to me in the whole animal kingdom. It forms the single exception to the most general rule amongst animals which possess a double heart. It accounts for the possibility of immersion in water or a noxious gas, without drowning or asphyxia; and it accounts for the possibility of a suspended respiration, without the feeling of oppression or pain, although sensation be unimpaired. It is, in a word, this peculiar phenomenon, which, conjoined with the peculiar effect of sleep in inducing diminished respiration in hibernating animals, constitutes the susceptibility and capability of taking on the hibernating state. On the other hand, as the rapid circulation of a highly arterialized blood in the brain and spinal marrow of birds probably conduces to their activity, the slow circulation of a venous blood doubtless contributes to the lethargy of the hibernating animal." 772.

6. When we turn to the *nervous system*, our author assures us that sensation and volition are quiescent, while the excito-motory, the true spinal system, retains its energy. Thus the slightest touch applied to one of the spines of the hedgehog immediately rouses it to draw a deep inspiration. The merest shake induces a few respirations in the bat. The least disturbance, in fact, is felt, as is obvious from its effect in inducing motion in the animal.

It is from misconception on this point that the error has arisen, that the respiration is not absolutely suspended in hibernation. This function has been so readily *re-excited*, that it has been considered as appertaining to the state of hibernation.

7. In the midst, says our able physiologist, of a suspended or partially suspended respiration, the *irritability* of the muscular fibre becomes proportionately augmenfed.

The single fact of a power of sustaining the privation of air, without loss of life, leads alone to the inference that the irritability is greatly augmented in the state of hibernation. This inference flows from the law already stated, and the fact. is one of its most remarkable illustrations and confirmations.

It might have been inferred from these premises, that the beat of the heart would continue longer after decapitation in the state of hibernation than in the state of activity in the same animal; an inference at once most singular and correct.

"This view," he continues, "receives the fullest confirmation from the following remarkable experiment: on March the 9th, soon after midnight, I took a hedgehog which had been in a state of uninterrupted lethargy during 150 hours, and divided the spinal marrow just below the occiput; I then removed the brain and destroyed the whole spinal marrow as gently as possible. The action of the heart continued vigorous during four hours, when, seeing no prospect of a termination to the experiment, I resolved to envelope the animal in a wet cloth, and leave it until early in the morning. At 7 o'clock A.M. the beat of both sides of the heart still continued. They still continued to move at 10 A.M., each auricle and each ventricle contracting quite distinctly. At half-after 11 A.M. all were equally motionless; yet all equally contracted on being stimulated by the point of a penknife. At noon the two ventricles were alike unmoved on being irritated as before; but both auricles contracted. Both auricles and ventricles were shortly afterwards unirritable.

This experiment is the most extraordinary of those which have been performed upon the mammalia. It proves several interesting and important points: 1. That the irritability of the heart is augmented in continued lethargy in an extraordinary degree. 2. That the irritability of the left side of the heart is then little, if at all, less irritable than the right,—that it is, in fact, veno-contractile. 3. That, in this condition of the animal system, the action of the heart continues for a considerable period independently of the brain and spinal marrow.

On April the 20th, at six o'clock in the evening, the temperature of the atmosphere being 53°, a comparative experiment was made upon a hedgehog in its state of activity: the spinal marrow was simply divided at the occiput; the beat of the right ventricle continued upwards of two hours, that of the left ventricle ceased almost immediately; the left auricle ceased to beat in less than a quarter of an hour; the right auricle also ceased to beat long before the right ventricle." 773.

Dr. Hall quotes from a paper of Mangili, in the Annales du Muséum, some further experiments strikingly corroborative of the preceding, and highly interesting iu themselves. We shall seize the opportunity of quoting them :—

"J'observai à peu près les mêmes choses dans une autre marmotte en léthargie, que je décapitai le 22 de Mars, 1807. Mais en ouvrant celle-ci, j'avois deux objets: le premier, d'examiner l'état des viscères les plus importans, comme le cœur, les poumons et le cerveau. Le second étoit de voir comment procédent les phénomènes de l'irritabilité musculaire; parce qu'ayant entendu dire à un célèbre naturaliste, que l'engourdissement avoit pour cause l'altération ou la suspension de cette irritabilité, il m'importoit de savoir si cette assertion étoit vraie. Dans la chambre où se trouvoit la marmotte, le thermomètre étoit à 6 degrés et demi; l'ayant introduit dans le bas ventre, il monta d'un degré, c'est-à-dire à 7 degrés et demi.

Je trouvai le les poumons dans leur état naturel. Le cœur continua à battre pendant plus de trois heures. Les pulsations, d'abord vives et frequentes, s'affoiblirent et se ralentirent peu-à-peu. J'en avois compté de seize à dix-huit par minute au commencement de la première heure ; à la fin de la troisième je n'en comptois plus que trois dans le même temps. Les veins du cerveau me parurent gonflés de sang.

La tête unie au cou ayant été séparée du tronc, je la mis dans un vase avec de l'esprit-de-vin, et j'y remarquai, même après une demi-heure, des movemens assez sensible. Ce fait prouve, ainsi que plusieurs autres dont je parlerai beintôt, que si dans l'état de léthargie conservatrice la vie est beaucoup moins énergique, le principe vital répandu dans les diverses parties, a beaucoup plus de tenacité, et tarde bien plus à s'éteindre.

Je séparai du corps de l'animal plusieurs morceaux des muscles qui obéissent à la volonté, et je vis avec étonnement que, trois heures après la mort, ils se contractoient fortement chaque fois que je les soumettois à l'action galvanique. Ces mouvemens convulsifs ne se ralentirent qu'au bout de quatre heures.

Il suit de là que les marmottes tuées pendant qu'elles sont en léthargie, présentent, relativement à l'irritabilité, à peu près les mêmes phénomènes qu'on remarque dans plusieurs animaux à sang froid.

Pour savoir ensuite si les phénomènes d'irritabilité étoient les mêmes dans l'état de ville et dans celui de léthargie, le 25 de Juin, j'ai fait périr, précisément de la même manière, une seconde marmotte qui étoit éveillée depuis deux mois, et qui faisoit de frequentes courses dans le jardin. Mon thermomètre marquoit ce jour-là 18 degrés : l'ayant introduit dans le ventre de la marmotte au moment où je venois de la décapiter, il s'éleva à 29 degrés.

Ayant mis le cœur à découvert, comme je l'avois fait dans mon expérience du mois de Mars, je comptai d'abord vingt-sept ou vingt-huit pulsations par minute. Ce nombre n'étoit plus que de douze au bout d'un quart d'heure, et de huit, au bout de demi-heure : dans les dix minutes suivantes, il n'y eut plus que quatre pulsations très-foibles par minute, et elles cessèrent totalement dans les dix derniéres minutes, c'est-à-dire cinquante minutes après la mort de l'animal ; tandis que le cœur de la marmotte tuée dans l'état de léthargie, donnoit encore quatre légères pulsations par minute, trois heures aprés que la tête avoit éte séparée du corps. Cette grande différence prouve que le principe de l'irritabilité s'accumule pendant la léthargie conservatrice.

Les chairs musculaires me semblirent plus pâles que celles de la marmotte en léthargie : elles etoient d'abord très sensibles à l'action galvanique ; mais ses signes d'irritabilité s'affoiblirent et disparurent bien plus rapidement. En effet les chairs musculaires de cette marmotte étoient peu sensibles au bout de deux heures, tandis que dans la marmotte tuée en hiver elles se contractoient fortement au bout de trois heures, et que l'irritabilité ne s'affoiblit notablement que quatre heures aprè la mort.

Les chairs des muscles intercostaux et abdominaux conservèrent leur sensibilité au stimulus électrique quelques minutes de plus que celles des membres ; d'où l'on peut conclure que le principe de l'irritabilité se conserve d'avantage dans certaines parties du même animal. Mais ce qui est prouvé jusqu'à l'évidence c'est que ce principe a bien plus de ténacité dans les chairs de l'animal tué pendant l'état de léthargie, que dans celle de l'animal tué pendant l'état de veille."

Dr. Hall remarks on this curious, though from what we know of amphibia and reptiles, natural fact, that its due value can only be determined by observing the dependence of the functions of life on that law of the inverse condition of the respiration and of the irritability, of which so much has already been said. In the hibernating animal the respiration is nearly suspended ; had not the irritability become proportionately augmented, the actions of life must have ceased !

8. The *motility* of the muscular fibre is unimpaired, Dr. H. assures us, in hibernation. If the hedgehog, he says, in a state of the most perfect lethargy, uncomplicated with torpor, be touched, its respiration is resumed, and it coils itself up more forcibly than before. The dormouse, in similar circumstances, unfolds itself; and the bat moves variously. Not the slightest stiffness is observed. The hedgehog, when roused, walks about, and does not stagger, as has been asserted. The bat speedily takes to the wing, and flies about with great activity, although exhaustion and death may subsequently result from the experiment. The phenomena are similar to those of awaking from natural sleep. Impaired motility, stiffness, lameness, &c. belong to torpor, and not to true hibernation.

3. *Of Reviviscence.*—Hibernation, observes Dr. Hall, induces a state of irritability of the left side of the heart, which, with high respiration and an arterialized blood, would be incompatible with life. Respiration suddenly restored, and permanently excited, is, therefore, as destructive as its privation in other circumstances.

" All those bats which were sent to me from distant parts of the country died. The continued excitement from the motion of the coach keeping them in a state of respiration, the animal perished. One bat had, on its arrival, been roused so as to fly about. Being left quiet, it relapsed into a state of hibernation. The excitement being again repeated the next day, it again flew about the room; on the succeeding day it was found dead.

It is in accordance with this law, that we observe hibernating animals adopting various measures to secure themselves from frequent sources of disturbance and excitement. They choose sheltered situations, as caverns, burrows, &c. secure from the rapid changes and the inclemencies of the weather and season. Many form themselves nests; others congregate together. The hedgehog and the dormouse roll themselves up into a ball. The common bat suspends itself by the claws of its hinder feet, with its head dependent, generally in clusters; the horseshoe bat *(ferrum equinum)* spreads its wings so as to embrace and protect its fellows.

All these circumstances are obviously designed to prevent disturbed hibernation.

In the depths of caverns, and other situations sheltered from changes of temperature in the atmosphere, the calls of hunger are probably the principal cause of reviviscence in the spring. The other causes of reviviscence are the return of warmth and external excitements: it is interesting to observe and trace the gradual return of respiration in the former case, and of the temperature of the animal in the latter.

If the hibernating hedgehog be touched even very gently, it draws a deep breath, and then continues to breathe for a short time. If this excitement be repeated, the animal is permanently roused, and its temperature raised. If the temperature of the atmosphere be augmented, the respiration is gradually excited, and the animal is gradually restored to its state of activity.

If a hibernating animal be excited in a very cold atmosphere, its temperature rises variously and then falls. A bat was perfectly lethargic in a temperature of 36°. A fine thermometer, with a cylindrical bulb, was introduced into its stomach; it rose to 39°. One hour afterwards, the animal not being further disturbed, the respiration was rapid, and the temperature in the stomach 95°. Shortly afterwards the temperature was 90°. The minute circulation was pretty good, and pulsatory in the arteries, the heart beating from twenty-eight to thirty-six times in the minute.

In another bat, in an atmosphere of the temperature of 36°, the thermometer in the stomach rose to 39°. The animal being continually excited, the temperature rose to 65°, but speedily fell to 60°.

The animal excited and revived in this manner is in a state of exhaustion and inanition. It is incapable of maintaining its temperature if exposed to cold, and will die unless it repass into a state of hibernation." 774.

It may, perhaps, admit of question whether it is the high irritability of the heart which kills because of its incompatibility with excited respiration and an arterialized blood. It is not proved that such irritability remains high when the respiration becomes high, nor is it clear why it should kill, if it did so. If a hibernating animal is stimulated to activity in a low temperature, it is reasonable to suppose that it would suffer, because it is unnaturally excited to exertions disproportionate to the hibernating state which such temperature tends to produce. If, on the other hand, the animal is stimulated in a higher temperature, it is not satisfactorily shewn that the excitation proves fatal, independently of the want of food, or an excessive quantum of excitation itself. We offer these not as objections but inquiries, and shall be glad to receive information from our able author. It is only fair to add a question of his own. The fact, he says, of the fatal influence of excited respiration during the augmented irritability of hibernation, contrasted with the similar fatal effect of suspended respiration, during the diminished irritability of the state of activity, will illustrate many of the causes, kinds, and phenomena of death. Do not these resolve themselves, in fact, into irritability insufficiently or excessively excited?

4. *Of Torpor from Cold.*—As Dr. Hall observes, it is important to distinguish that kind of torpor which may be produced by cold in any animal, from true hibernation, which is a property peculiar to a few species. The former is attended by a benumbed state of the sentient nerves, and a stiffened condition of the muscles; it is the direct and immediate effect of cold, and even in the hibernating animal is of an injurious and fatal tendency; in the latter, the sensibility and motility are unimpaired, the phenomena are produced through the medium of sleep; and the effect and object are the preservation of life.

True hibernation is induced by temperatures only moderately low. All hibernating animals avoid exposure to extreme cold. They seek some secure retreat, make themselves nests or burrows, or congregate in clusters, and, if the season prove unusually severe, or if their retreat be not well chosen and they be exposed in consequence to excessive cold, many become benumbed, stiffened, and die.

Dr. Hall continues :—

"To induce true hibernation, it is quite necessary to avoid extreme cold; otherwise we produce the benumbed and stiffened condition to which the term torpor or torpidity may be applied. I have even observed that methods which secure moderation in temperature, lead to hibernation: hedgehogs, supplied with hay or straw, and dormice, supplied with cotton-wool, make themselves nests and become lethargic; when others, to which these materials are denied, and which are consequently more exposed to the cold, remain in a state of activity. In these cases, warmth or moderated cold actually concur to produce hibernation.

When we read of insensibility, of a stiffened state of the muscles, and of a cessation of the circulation, as obtaining in hibernation, we may be certain that a state of torpor has been mistaken for that condition. The actually hibernating animal exposed to continued severe cold is, as M. Saissy correctly observes, first roused from this state of ease and preservation into a painful activity, and then plunged into a fatal torpor." 775.

He adds:—

"In conclusion, one of the most general effects of sleep is to impair the respiration, and with that function the evolution of animal temperature. The impaired state of the respiration induces a less arterial condition of the blood, which then becomes unfit for stimulating the heart; accumulation of the blood takes place in the pulmonary veins and left auricle; a sense of oppression is induced, and the animal is either roused to draw a deep sigh or awakes altogether.

Such are the phenomena in animals in which the heart has not the faculty of taking on an augmented state of irritability, with this lessened degree of stimulus. But in those animals which do possess this faculty, a property which constitutes the power of hibernation, the heart continues the circulation of the blood, more slowly indeed, but not less perfectly, although its arterial character be diminished, and its stimulant property impaired. No repletion of the pulmonary veins and of the left auricle, no sense of oppression is induced, and the animal is not roused; the respiration continues low, the temperature falls, and the animal can bear, for a short period, the abstraction of atmospheric air.

All the phenomena of hibernation originate, then, in the susceptibility of augmented irritability. The state of sleep, which may be viewed as the first stage of hibernation, induces an impaired degree of respiration. This would soon be attended with pain, if the irritability of the heart were not at the same time augmented, so as to carry on the circulation of a less arterial blood, and the animal would draw a deep sigh—would augment its respiration or awake. Occasional sighs are, indeed, observed in the sleep of all animals, except the hibernating. In these, the circulation goes on uninterruptedly, with a diminished respiration, by the means of an augmented irritability. There is no stagnation of the blood at the heart; consequently, no uneasiness; and the animal becomes more and more lethargic, as the circulation of a venous blood is more complete. This lethargy is eventually interrupted by circumstances which break ordinary sleep, as external stimuli or the calls of appetite." 776.

Our author enumerates the hibernating animals. The most unequivocal are the bat, the hedgehog, the marmot, the hamster, the dormouse. It has been said that the bear and beaver belong to the number, but this is extremely doubtful. It has been said also that the swallow belongs to the hibernating class, but this is incorrect. The cold-blooded animals, the Chelonian, the Saurian, the Ophidian, and the Batrachian tribes, all, however, indubitably pass the winter in a state of apathy and lethargy. Some of the fishes also become lethargic during the cold season. The same remark applies to some of the molluscous and insect tribes.

We cannot but think Dr. Hall's paper highly interesting. It displays his aptitude for clear and discriminating generalizations, and for resolving into simple formulæ many and apparently contradictory facts.

### II.—ON ANIMAL HEAT.

THE fifteenth and sixteenth Parts of the Cyclopædia contain an article on this subject by Dr. W. F. Edwards. Some observations on it will also be found in Müller's Physiology, now in process of translation by Baly; and we propose to abstract from both, but more particularly from the first, a consistent account of what is at present known on this most important physiological problem. A correct acquaintance with the laws of animal heat will tend materially to improve our practice, and by opening to us philosophical and extended views of the economy, will especially enable us to appreciate more satisfactorily those influences of climate and of season, which affect so vitally the human race. We would direct the attention of our readers to the inquiry, which has a very direct and essential bearing on the maintenance of health and the treatment of disease.

Dr. Edwards commences by remarking, that it was the natural but erroneous supposition, before the application of the thermometer, that the temperature of our bodies is continually varying. The thermometer corrected that mistake but bred another—that our temperature does not vary at all. Still, he says, the measures of temperature given by different observers did not perfectly accord, though each presented his conclusions as the temperature of the race. It was but reasonable to imagine that these discrepancies arose not from any want of accuracy in observation, but from diversities inherent in the subjects observed. This is now known to be the case. But though proofs of this truth have been greatly multiplied, the whole subject has never been presented in a connected and systematic manner.

Since it is proved that the temperature of the human body varies, we can only obtain an approximation to its actual amount by taking the mean of all the good observations that have ever been made, being particularly careful to include the extremes; for a mean gives but a very imperfect idea of a term that ought to represent a variable number, if the limits are not at the same time assigned and taken into the account. The best observations of this kind, provided they be sufficiently numerous, will be those that have been made by the same individual, inasmuch as there is great likelihood that he will always have made use of the same procedure and of the same instruments, by which the results become more readily comparable one with another.

*Temperature of the Human Body.*—Dr. Edwards employs the measures of temperature given by Dr. John Davy. They amount to one hundred and fourteen in number, and were made on individuals of both sexes and of different ages in three quarters of the world, in Europe, Asia and Africa, in different latitudes, under various temperatures, and among individuals of different races.

The mean age of the subjects of Dr. Davy's observations was twenty-seven years. The mean temperature of the air was 23°, 3 c. (74° F.*) between the limits of 15°, 5 (60° F.) and 27°, 8 (82° F.). In these circumstances the mean temperature of the body, which was always taken in the mouth, was 37°, 7 (100° F.) between the extremes 35°, 8 (96°, 5 F.) and 38°, 9 (102° F.). The greatest difference in one hundred and fourteen ob-

* "[The valuations according to Fahrenheit's scale the editor desires may be regarded as mere though close approximations to the indications according to the centigrade scale.]—Ed."

servations, therefore scarcely exceeds three degrees. The temperature of the human body thus obtained might be considered as exact if the conditions of age and external atmospheric temperature approached pretty closely to their respective means. This, in fact, was the case as regards the first term, but not as concerns the second; for some of the observations were made under very intense degrees of heat, such as 27°, 8 (82° F.), but none at the opposite extreme, or at a temperature which could be reputed cold, a temperature of 15° (59° F.) being already sufficiently agreeable. So that if the temperature or the air influences that of the body, the mean which has been stated as the temperature of the species would be too high.

It is, of course, requisite, in order to enlarge our notions and exclude sources of fallacy, to examine the temperature of other animals, as well as that of man.

*Temperature of the Mammalia.*—The observations here (we quote and shall quote Dr. Edwards) were made on thirty-one different species taken from among the principal divisions of this class, and under a mean temperature of the external air equal to 25° (77° F.) between the limits of 15° (59° F.) and 30° (86° F.).

The mean temperature of the body of the Mammalia was 38°, 4 (101°, 10 F.), the maximum being 40°, 5 (105° F.), the minimum 37°, 2 (99° F.), The extent of variation consequently presented by the Mammalia, 3°, 3 of the centigrade scale (6° F.), is nearly equal to that exhibited by man. But there is this difference between the two scales, that the extremes and the mean in the case of man are inferior to the corresponding terms in the case of the Mammalia.

*Temperature of Birds.*—The observations here were made on fifteen species in different orders. The mean temperature of the air was 26°, 1 (79° F.), between the extremes 15° (59° F.) and 31°, 5 (88°, 75 F.). The temperature of the subjects of the experiments offered a mean of 42°, 1 (170°, 86 F.), the superior limit being 43°, 9 (111° F.), the inferior 37°, 2 (99° F.) The temperature of birds, therefore, presents a scale much more extensive than that of man and the Mammalia, amounting to as many as 6°, 7 degrees centigrade (12° F.). It also stands above both of the others in the point of its mean, which is higher by 3°, 7 (6° F. nearly) in its upper limit, and 5° centigrade (about 9° F.) higher in its lower limit. The lower limit, in fact, corresponds very nearly with the mean term of the heat of the Mammalia as exhibited in the preceding scale. Much cannot, however, be said on the minimum of heat in these instances, no observation having been taken at a temperature lower than 59° F.

*Temperature of Reptiles.*—From nine observations made on members of the four orders of Reptiles, Dr. Davy found, the external air having a mean temperature of 26°, 5 (79°, 75 F.) between the extremes 32° and 16° (89°, 5 and 60°, 75 F.), that the temperature of Reptiles was not higher than 28° (82°, 5 F.).

*Temperature of Fishes.*—If from Reptiles we pass to Fishes, corresponding and even more remarkable differences are perceived. Dr. Davy, indeed, gives the temperature of no more than five species, but they belonged to very different orders. The mean external temperature being 22°, 3 (72° F.), that of fishes was found to be but 23°, 2 (74° F.) which is very little more than one degree centigrade higher. This difference becomes even more striking, if possible, as we descend in the scale of animals.

*Temperature of Insects.*—From eight observations on insects of very dissimilar species, the mean temperature of the air being 24° (75°, 5 F.), that of the insects was 24°, 2 (75°, 75 F.)

*Temperature of the Crustacea.*—Two species of Crustaceans, the cray-fish and crab, presented a still more interesting phenomenon. The mean temperature of the air was 24°, 4 (76° F.) at the time of experimenting, that of the Crustacea 24°, 1, or somewhat lower than the ambient medium. This we do not presume to present as the rule, but we would say that the temperature of the Crustacea is nearly equal to that of the medium in which they are plunged.

*Temperature of the Mollusca.*—In observing the temperature of a single Mollusc, the common oyster, the temperature of the sea being 27°, 8 (82° F.) that of the animal was 27°, 8 also.

It is obvious that, in regard to temperature, animals, from reptiles inclusively downwards, may be arranged in a single group and termed *cold-blooded;* while mammalia and birds will constitute a higher class, the *warm-blooded.*

To characterise the temperature of the latter great division, the mean of the temperatures of the respective classes which compose it must be taken first. From the experiments of Dr. Davy the mean temperature of mammalia appears to be........................... 38°, 4 (101° F.)
that of birds ................................ 42°, 1 (108° F.)

Mean of both classes............ 40°, 25 (104°, 5 F.)

We may therefore say that the mean temperature of warm-blooded animals, including man, surrounded by a moderate external temperature is in round numbers 40° (104° F.) between the limits of 36° and 44° (97° and 111°, 5 F.), by which we have a scale of difference amounting to 8° (about 14° F.).

The other class, that, namely, including the *cold-blooded animals,* having no peculiar temperature proper to them, may be characterized in the following manner:—their temperature differs little or not at all from that of the surrounding media in which they live, when this is at a degree which may be called moderate.

*General Conditions of Organization relatively to the Production of Heat.*—So marked a difference existing in the temperature of the two divisions of animals, it is a natural presumption that some grand differences exist also in their structure. And such is found to be the fact. It is a natural presumption too, that if such differences of structure and of temperature coincide, they have the mutual relations of cause and of effect. To determine whether this presumption is correct it is necessary to pursue the train of investigation which is found to succeed to in the great divisions of natural science—to exclude certain phenomena from the mass to be examined and watch the effects of such exclusion—to insulate, in fact, particular phenomena and observe their actions and their habitudes when uncombined and unimpeded with those of others. Nature helps us by the diversity of the forms of animal life which she disports in, and we must help ourselves where she does not go quite far enough, by special experiments of our own.

Let us apply then this philosophical method to the case before us. We have said that the warm and the cold-blooded animals, differing as they do, differ also in structure;—what *are* those latter differences?

1. Mammalia and birds have the double circulation, which the cold-blooded have not.

2. The character which distinguishes the respiratory organ of warm-blooded animals from that of cold, is this,—that either in itself or its appendices (the air-sacs of birds) it presents a much larger extent of surface in relation with the air.

3. Warm-blooded animals are farther distinguished from the cold-blooded by important modifications of the digestive canal. 1. The first portion of the apparatus from the mouth to the stomach is much more complex in them; for instance, it presents either a much more perfectly developed dental system, fitted to divide the food, or a sac, as among birds, fitted to macerate the aliment, and cause it to undergo a kind of preparatory digestion before it is passed to the stomach. 2. The stomach is more distinct; either the entrance to and exit from this pouch are better marked, being often provided with a valve, as in the mammalia, or its structure and form are more special, as we observe it among birds. 3. The intestinal canal is much longer in the warm than in the cold-blooded tribes.

4. The nervous system presents diversities still more important and well-marked. The most striking character exists in the proportion of the principal trunk of this system, and especially of its encephalic extremity, which is much larger in the warm than in the cold-blooded animals.

These organic conditions, which are mutually dependent, may be reduced to the expression of these two general ones:—1st, the formation and distribution of the arterial blood, the particularly exciting and nutritive blood of the body; 2nd, the most powerful influence of the nervous system.

As these are almost the sole points of any consequence that distinguish the structure of warm and of cold-blooded animals, it follows, that, within the circle which they form, the causes of difference of temperature must lie.

"Conditions of organization and of functions may be entitled the physiological causes of the production of animal heat. If we succeed in determining these we ought to rest satisfied. If, indeed, to this knowledge we could add that of the immediate cause of this phenomenon among animals, or what is the physical cause, it would be a great gain for science. This, accordingly, was the object of the labours of the majority of physiologists who have given their attention to the subject of animal heat. But they could not possibly succeed in their researches, for the simple reason that natural philosophers themselves have not yet discovered how heat is produced in the inorganic world; although indeed they have presumed that they were acquainted with it." 651.

The immediate cause of animal heat is a problem not likely to be solved. But we may connect it with some particular function or functions, and having done so much we shall do well. We must proceed in the manner we have already mentioned, varying the conditions under which heat is produced, either by our own experiments, or by availing ourselves of the innumerable experiments ready made for us by nature.

Mammalia and birds have been shewn to have a higher temperature than reptiles and fishes, and the conditions which distinguish the two classes have been stated generally. The next thing is to compare the orders with each other, and observe the distinctive characters of each. Compare then mammalia and birds.

The lungs of birds, writes Dr. Edwards, although smaller, are more loaded with blood than those of the Mammalia, and are in communication with extensive air-cells, spreading all through the body and even penetrating into the cavities of the bones, so that the air may be said to penetrate the body generally, and to be in contact with the ramifications of the aorta as well as with those of the pulmonary artery; the blood of these animals is therefore in the most extensive relation imaginable with the air of the atmosphere. Again, if the nature of the blood of Birds be considered, independently of this extensive relation with the air, the organic condition here will not appear less favourable to them. The globules of this fluid, indeed, are a little larger and less spherical than in Mammalia, which is a disadvantage; but the proportion they bear to the fluid part is so favourable to birds that this circumstance must give them immensely the advantage in reference to the character which engages us. With regard to the nervous system, if the encephalic extremity is developed in a minor degree in birds, their circulating and respiratory systems act with greater quickness. Lastly, and as an effect of the whole of these conditions, the consumption of air is much greater among birds than among Mammalia.

The respiratory system then, is more developed in birds than in mammalia —the nervous less. The mean temperature of the mammalia is 38°, 4 (101° F.), that of birds 42° 1 (108° F.).

Compare next reptiles with fishes.

The digestive apparatus is more complete among reptiles than among fishes; 1st, in the dental apparatus when it exists; 2d, in the more distinct stomach; 3rd, in the greater length of the intestines.

The blood of reptiles is superior to that of fishes both as regards the nature of the globules and their relative proportion, their size being smaller and their numbers greater, than among fishes.

If the whole of the blood in the reptile is not transmitted through the organ of respiration, whilst in the fish it is, a larger quantity of this fluid is brought into contact with the air in the same space of time in consequence of the greater extent of surface of the organ in the reptile, and then the reptile has the farther immense advantage of a pulmonary or aerial respiration, whilst that of the fish is branchial or aquatic. To conclude, the nervous system of reptiles is much more developed in the cerebro-spinal axis, and especially in the encephalic extremity, than in fishes.

Perhaps the most striking difference between reptiles and fishes is the fact, that the first respire in air, the latter in water. The development of heat is greater in reptiles than in fishes.

To pursue the inquiry, is to compare the cold-blooded vertebrata with the invertebrata.

"A glance suffices to shew the vast inferiority of the invertebrata in this as in every other respect. In the first place their blood is so little of the same nature as that which has been recognised most favourable to the production of heat, that it wants the characters whether of arterial or of venous blood. The blood of the invertebrata, with the exception of a very small group (the worms with red blood), is colourless. In the structure and number of its globules it is also greatly inferior. The globules, indeed, may be smaller, but then they are of a much more simple structure, and consequently lower in the scale, in other words, more imperfect. In the relation to the fluid part of the blood too, they are in much smaller proportion than among the vertebrata. An analogous character is manifest in the tissues generally, the proportion of water in them being incomparably larger in the invertebrate than in the vertebrate series of animals. Finally, there is an immeasurable inferiority in the nervous systems of the invertebrate compared with even the lowest of the vertebrate series of animals." 653.

The invertebrata have a much smaller capability of producing caloric than the cold-blooded vertebrata.

"The comparison might be carried out in regard to insects and the mollusca, which present some appreciable differences. If attention were confined solely to the structure of the greater number of the organs of nutrition, which are much more largely developed in molluscs, it might be inferred that they had a higher caloric power than insects; but when we take into the account, 1st, the final result of the nutritive functions, the quality of the tissues, which in the mollusc are much more loaded with watery fluid, by which they acquire a greater degree of softness and flaccidity, (whence the class has its name,) whilst in the insect they are, on the contrary, as remarkably dry and firm;—2d, when the most general mode of respiration is compared in the two divisions, it being in the insect aerial, in the mollusc aquatic; 3d and lastly, when we glance on the one hand and on the other at the state of the nervous system, and observe how much less perfectly this is developed in most of the molluscs than in the insect, it is impossible not to perceive that according to the principles influencing the production of heat, the mollusca must be inferior in this respect to insects. This is indeed the result of observations of all kinds, however imperfect or limited these may have been." 654.

It is impossible, for very intelligible reasons, to carry the investigation and comparison lower down in the scale. The phenomena connected with heat in the lower grades of the animal creation become inappreciable; and this even in virtue of the same principle that has been announced; for the tissues are found to become more and more watery as we descend in the scale, till at length the solid constituent is almost inappreciable. Of course the circulating fluid must be watery in a still greater ratio; it contains but few globules; and then the nervous system falls off in a still greater proportion; it becomes more and more imperfect, till at length no trace of it is to be discovered.

The general temperature of animals has been examined. The next point is to ascertain the

*Temperature of different Parts of the Body.*—Our own feelings tell us that different parts of the body have different temperatures. A law of decrease of temperature in the ratio of the distance of parts from the heart had even been deduced from observations of that kind. But when exact measurements came to be taken this law was soon found to be at fault. Dr. Davy, in taking the temperature of the different parts of the body of a lamb, found that of the right ventricle of the heart 40°, 5 (105° F.), that of the left ventricle 41°, 1 (106° F.) The left ventricle was therefore higher in temperature than the right, to the extent of a degree of the scale of Fahrenheit's thermometer. The temperature of the rectum corresponded with that of the right ventricle.

In my enquiries, says Dr. Edwards, along with M. Gentil into the relations in point of temperature of certain external parts, we found in a strong man, perfectly at rest in mind and body, in the month of July, the external air being at 21°, 25 c. (71° F.), the temperature of the mouth 38°, 75 (102° F.) ; that of the rectum corresponded. The hands presented the next highest degree, marking nearly 37°, 5 (99°, 5 F.) What is remarkable is that the axillæ and groins, which corresponded with one another, were very sensibly lower in temperature than the hands ; they did not raise the thermometer higher than 36°, 96 c. (99° F.). The cheeks marked 35°, 93 (96°, 5 F.), the temperature being ascertained by enveloping the bulb of the thermometer in the skin of these parts. The feet were a little lower, 35° 62 (about 96° F.) ; their temperature being determined by placing the thermometer between the two, so that the bulb was surrounded on every side. The temperature of the feet was, therefore, notably lower than that of the hands, differing to the extent of 1°, 88 of the centigrade scale (above 3° of Fahrenheit's thermometer). Placed on the skin between the thorax and abdomen the thermometer was at its minimum, not rising higher there than 35° (95° F.) ; but here a part of the bulb being in contact with the air, there must have been considerable cooling.

Internal parts seem to have some such relations of temperature with each other as the following :—

1st. The warmest part of the body, according to John Hunter, is in the abdomen, close to the diaphragm. 2d. The next part in point of temperature is the left ventricle of the heart. 3d. The right ventricle of the heart is the next in succession. The rectum and the mouth shut are of the same temperature. The greatest difference consequently between the temperature of these internal parts does not amount to more than 1° centigrade, or at the utmost 2° Fahrenheit.

Supposing the relations in temperature of the internal parts to be pretty constant in the normal state, the temperature of the right ventricle of the heart and of the rectum may be determined by taking the temperature of the closed mouth ; that of the left ventricle will be found by adding 0,44 c., or 1° F. to the degree indicated.

*Relations in point of temperature between external parts.*—We have only data for instituting comparisons in regard to the hand, the axilla, the groin, and the feet, among the external parts of the body. In a moderate summer-heat the hand appears to be the part which is most susceptible of showing a high, the feet the parts most susceptible for exhibiting a low temperature. The axilla and groin generally exhibit nearly the same degree of temperature ; and the amount in which they differ in this particular from the mouth may be stated at about 1°, 75.

But the thermometer is, for obvious reasons, a coarse and an imperfect instrument for ascertaining the temperature of the deeper parts. These considerations, says our author, led to the adoption of thermo-electrical means by Messrs. Becquerel and Breschet. The processes they employed in procuring indications of temperature were the following. The only means we have of penetrating into the interior of organs without injury is to make use of a needle similar to that employed in acupuncture. Now it is easy to arrange this needle so as to obtain thermo-electric indications, which proclaim immediately and with the greatest precision the temperature of the part or medium with which the point happens to be in contact. It is enough to compose this needle of two others in metal, two of the extremities of which are soldered together in a few points only, whilst the other two are placed in communication with one of the extremities of the wire of an excellent thermo-electric multiplier. The slightest changes of temperature at the points of junction give origin to an electrical current, which, in reacting on the magnetic needle, causes it to deviate by a certain number of degrees, which consequently become indices of the temperature of the point of the needle, and therefore of the medium in which it is placed, The multiplier ought to be so sensitive as to show a deviation of one degree of the magnetic needle for each one-tenth of a degree of temperature as measured by the centigrade scale, an amount of temperature made sensible by the union of the two ends of the wire which forms its circuit with an iron wire soldered by its ends.

Dr. Edwards relates a series of experiments too circumstantial for our purpose. The main fact is that muscular contraction developes heat in the muscle and its vicinity, chiefly, no doubt, from the afflux of blood to them. Dr. Edwards remarks :—

"In the rise of temperature observed along with muscular contraction, we have in the first place only considered the action of the blood; but neither contraction of the muscles nor the afflux of a larger quantity of blood could take place without the nervous influence; for it is the will which determines the muscular contraction, and the will only acts through the medium of the nerves which are distributed to the muscles. From this consideration it follows equally as from general relations previously exposed, that whatever lessens the nervous influence will likewise tend to reduce the temperature. Here we are, then, reverting to the two general conditions which we had already found to be the most influential in calorification, namely, the arterial blood and the nervous system." 658.

*Influence of External Temperature.*—The source of heat being in animals in the centre, it is reasonable to suppose that the surface would be influenced by the lower temperature of the surrounding medium, and be rather cooler in consequence. And such is found to be the case. If the external temperature should vary, it is also natural to suppose that the temperature of the surface of the animal would vary too. This is found to be the case also, but not to any considerable amount. Dr. Davy made the following series of observations :—

| *Temperature of the air.* | *Mean temperature of five men.* |
|---|---|
| 15°, 5 (60° F.) | 36°, 85 (98° F.) |
| 25°, 5 (78° F.) | 37°, 16 (99° F.) |
| 26°, 4 (79° F.) | 37°, 32 (99° 5, F.) |
| 26°, 7 (80° F.) | 37°, 58 (100° F.) |
| 27°, 8 (82° F.) | 37°, 70 (100° 5, F.) |

The changes in the temperature of the body in these cases must be admitted to be slight, for whilst the temperature of the air varied to the extent of 12°, 3 c., the changes in the mean temperature of the body did not exceed 0°, 9. The results, however, are confirmed by the observations of Dr. Reynaud, surgeon of the corvette La Chevrette, in a voyage of discovery in the Asiatic seas, undertaken in the year 1827.

| | |
|---|---|
| Mean temperature of the body deduced from observations four times repeated upon each of eight men, under the torrid zone, the external temperature varying from 26° to 30° c. (79°, 86° F.) | 37°, 58 (100° F.) |
| Mean temperature of the same eight men observed three times in the temperate zone, the external temperature varying from 12° to 17° (53° to 62° F.) | 37°, 11 (99° F.) |

It is to be regretted that these experiments have not been carried into low temperatures. But in the voyage of Capt. Parry it was observed that the temperature of the mammalia was very high. With the external thermometer at — 29°, 4 (— 21°, F.), the temperature of the white hare was + 38°, 3 (101° F.) With the thermometer at — 32°, 8 (27° F.), the temperature of a wolf was + 40°, 5 (105° F.) : the temperature of the Arctic fox, under nearly the same circumstances, namely, when the thermometer was standing at — 35° (— 31° F.), was as high as + 41° 5 (107° F.). Similar observations have since been made in the same high latitudes upon man.

Dr. Edwards has studied and experimented on the influence of seasons on the temperature of warm-blooded animals. The observations made on sparrows exhibited the greatest differences. In the month of February the mean temperature of these birds was found to be 40°, 8 (105° F.); in April 42° (108° F.), in July 43°, 77 (111° F.) The temperature from this time began to decline, and followed, in the same ratio in which it had increased, the sinking temperature of the year.

It is shewn to be the fact, what our sensations would indeed make us believe to be so, that water both cools and heats far more than air. And, from immersions of parts of the body only in water, the following deductions may be drawn :—1st, that partial chills, or the exposure of individual parts to low temperatures, may be and are felt very extensively even when the cold is not very severe ; 2nd, that the chilling of a single part, such as the hand or the foot, may cause a loss of temperature in all the other parts of the body, even far beyond what could have been presumed as likely or possible. These facts give a key to the right understanding of the immense influence which partial chills are capable of exercising on the state of the general health.

*The Effects of partial Heating* are not altogether unimportant. The hand being immersed in water heated to the temperature of 34° R. (109° F.), rose one degree of the same scale, and the temperature of other remote parts not immediately exposed to the influence of heat were found to have risen in a corresponding degree. Whence follows this axiom,—*that we cannot either raise or lower the temperature of any one part of the body without all the other parts of the frame being affected, and suffering a corresponding rise or fall in temperature, more or less according to circumstances.*

*Effects of a very high or a very low external Temperature on the Temperature*

*of the Body.*—On a summer's day, the temperature of the air being 37°, 77 c. (100° F.), Franklin observed that the temperature of his own body was nearly 35°, 55 c. (96° F.) This fact proves, of itself, that man has the power of keeping his temperature inferior to that of the air. In numerous experiments made in England by Dr. Fordyce and his friends, and subsequently by Dr. Dobson, in which these experimenters exposed themselves to very high temperatures, which on some occasions exceeded that of boiling water, the heat of the body was never observed to rise more than one, two, three, or four degrees of Fahrenheit's scale at the utmost. The highest temperature of the body noticed by Dr. Dobson is 102°.

"The highest temperatures of the human body exposed to excessive heats ever observed, were remarked by Messrs. Delaroche and Berger in their own persons. The temperature of M. Delaroche being 56° 56 c. (98° F.) increased 5° of the centigrade scale, by remaining exposed in a chamber the temperature of which was 80° c, (176° F.). M. Berger, whose temperature was the same as that of M. Delaroche, gained 4° c. by remaining for sixteen minutes in the hot chamber at 87° c. (188°, 5 F.). These experiments are liable to this objection, that the temperature was taken in the mouth in an atmosphere of much higher temperature, which might have some influence in raising the thermometer. To arrive at conclusions against which no kind of objection could be raised, Messrs. Delaroche and Berger exposed themselves in succession in a box, out of which they could pass their head; the hot air or vapour of the interior being prevented from escaping by means of a circular pad of soft napkins placed between the edge of the outlet and the neck. The temperature of the mouth, in this way, if it was increased, must be increased in consequence of a rise of temperature in the parts of the body included in the bath. After a stay of seventeen minutes in the bath, heated from 37°, 5 to 48°, 75° c. (99° to 120° F.), the temperature of M. Delaroche's mouth rose 3°, 12 c. Under similar circumstances, the temperature of the bath being from 40° to 41°, 25 c. (104° to 106° F.), the temperature of M. Berger's mouth increased 1°, 7 c. in the course of fifteen minutes.

It is pretty obvious that experiments upon the human subject cannot be pushed far enough to ascertain the highest amount of temperature that can be acquired under the influence of exposure to air of excessively high temperature. To judge of this analogically, recourse must be had to warm-blooded animals of the two classes, mammalia and birds. Messrs. Delaroche and Berger consequently exposed different species of mammalia and birds to dry hot air of different temperatures, from 50° to 93°, 75 c. (122° to 201° F.), leaving them immersed till they died. The whole of the animals that were made subjects of experiment, in spite of diversity of class and species, and of the varieties of temperature to which they were exposed, had gained an increase of temperature nearly equal at the moment of their death. The limits of the variations being between the terms 6°, 25 and 7°, 18 c., the amount of difference did not exceed 0°, 93 c. which is a very trifling quantity. It may therefore be inferred, that man and the warm-blooded animals cannot, under the influence of exposure to dry air of excessively high temperature, have the heat of their body raised during life to a greater extent than from 7° to 8° c. The temperature of the body being increased to this extent becomes fatal. It is in fact only attained at the moment of dissolution; perhaps death has virtually taken place before it is attained." 661.

The cold-blooded animals exhibit during the heats of Summer a temperature inferior to that of the air. Thus the temperature of the atmosphere being 32° c, (90° F.), that of a tortoise was only 29°, 4 (85° F.). And many such experiments might be reported.

It is scarcely necessary to observe that evaporation is a cooling process to animals, and that it acts in a very efficient manner. We pass to the :—

*Relations of the Bulk of the Body to Animal Heat.*—"If the temperature of the larger animals be compared with that of the smaller, it will be found that the former do not mark so high a degree as the latter. In the elephant and horse foi instance, no higher a temperature than 37°, 5 c. (100° F,) has been observed, whilst in the rat and squirrel temperatures of 38°, 8, and of 39°, 4 (102° and 103° F.) have been noted. To prove that the difference is less owing to the order or species than to the simple size, we shall contrast several animals belonging to the same order, selecting the ruminants. The temperature of the air being the same, namely, 26° c. (79° F.), the temperature of the ox was found to be 38°, 9 (102° F.), whilst that of a castrated he-goat was 39° 5 (103° F.), and that of the she-goat and sheep 40° (104° F.)." 662.

Smallness of size must, per se, tend to lower temperature from the comparatively great amount of surface exposed to the inferior temperature of the air. But, on the other hand, the circulatory and respiratory motions are more rapid in animals of small than of greater bulk, and that is a circumstance favourable to the production of heat.

*Relations of Age to Animal Heat.*—It is certainly of consequence to ascertain these. Dr. Edwards has made some experiments which throw much light upon them.

"If," he says, "the temperature of new-born puppies lying beside their mother be taken, it will be found from one to three degrees inferior to that of the parent. The same thing obtains in regard to the young of the rat, the the rabbit, the guinea-pig, &c. and is probably universal among the mammalia. Among birds the same circumstance presents itself in a still more marked degree. If they be taken out of the nest in the first week or even fortnight of their existence, the difference of temperature extends to from 2° to 5° c. between the young and the parents. The fact has been ascertained in regard to the sparrow, the swallow, the martin, the sparrow-hawk, the magpie, the thrush, the starling, &c. &c., and is probably, as among mammalia, universal. Whence we may conclude that the phenomenon is general as regards warm-blooded animals. We might have taken it for granted that man was comprised within the category, but it is just as well to have the assurance that he forms no exception to the law, that he has no peculiar privilege in this respect. To have a precise term of comparison, the temperature of twenty adults was taken at the same time, the thermometer being applied in the axilla. The temperature of these twenty persons varied between 35°, 5 and 37° c. (96° and 99° F.) ; the mean term was therefore 36°, 12 (97° F.). The temperature of ten infants varying from a few hours to two days in age, ascertained in the same manner, varied between 34° and 35° 5 c. (93°, 5 and 96° F.). The mean was therefore 34°, 75 c. (about 94°, 5 F.). There was consequently a difference of nearly two degrees between the temperature of the adult and of the newly-born babes. Man is therefore proved to be subjected to the same law here as animals having warm blood in general, the young of which, so far as they have been examined, and we may presume universally, are inferior in temperature to their parents." 663.

But if the manner of experimenting is altered, results of an extraordinary character come out. To develope these the temperature of the newly-born being must not be taken only when it is in contact with its mother. If, after having ascertained the temperature of a puppy in this position, it be removed from the mother and kept isolated, the temperature will be found to fall rapidly; and this phenomenon takes place not only when the air is cold, but when it is mild. The phenomenon does not commence after a term; it is apparent from the moment the separation takes place, and is very sensible after the lapse of a few minutes. The following is the rate of cooling of a puppy twenty-four hours old, the external temperature being 13°, c. (about 55° 5 F.), taken at intervals of ten minutes; the series of course represents the successive losses of temperature in the course of the small intervals of time indicated:—temperature in commencing the observations 36°, 87 c.; the declensions in temperature at intervals of ten minutes successively, 0°, 63, 1°, 12, 1°, 38, 1°, 25, 1°, 29, 0°, 87, 1°, 63, 0°, 25, 1°, 0; in thirty-five minutes the temperature declined farther 1°, 25; in thirty-five minutes more it fell 3°, 12; in thirty minutes more 2°, 50; in twenty-five minutes more 1°, 25; in thirty minutes more 1°, 25; so that in the course of four hours in all, the temperature declined by the amount of 18°, 12 of the centigrade scale (about 33° F.)! Not only had the temperature of the animal sunk by so large a quantity in so short a period of time, the external temperature being pleasant, but it actually could maintain its temperature at no higher a grade than 6°, 75 c. (14°, 5 F.) above that of the atmosphere. Experiments of the same kind performed on three other puppies of the same litter presented results in all respects analogous. The cooling may even go much further by protracting the period during which the young animals are kept apart from their parent. For instance, four puppies, twenty-four hours old and of much smaller size than the subjects of the former experiments, after having sunk 16° c. in four hours and thirty minutes, lost six degrees more of temperature in the succeeding eight hours and thirty minutes, the air remaining all the while at 13° c. (55°, 5 F.) They consequently lost twenty-two degrees centigrade in thirteen hours; and, what is very remarkable, their final temperature was but one degree above that of the surrounding air. Kittens and rabbits of the same age exhibited similar phenomena, if possible in a more striking degree. Some kittens were observed to cool twenty degrees centigrade within the short interval of three hours and a half, and some young rabbits suffered the same depression of temperature in two hours and ten minutes, the air being at the time at 14° c. (57°, 5 F.).

This does not, however, hold with all animals, and Dr. Edwards gives us a formula for those with whom it does and does not obtain.

*Those species of mammalia which in the earlier period of their existence do not maintain their temperature, that of the external atmosphere being mild or warm, but cool down to the standard of the cold-blooded animals, are born with their eyes closed; whilst those which maintain their temperature, that of the external atmosphere being mild, are born with their eyes open;* and this, whether they can walk about like the guinea-pig, the kid, &c. or cannot do so, as is the case with the human infant in particular.

Dr. Edwards observes, that, in puppies, at the period of their birth, the ductus arteriosus continues pervious and of large size. The consequence of this structure is that a free communication is established between the arterial and venous blood, by which they are mingled in large proportion one with another. And here we have precisely the physiological character derived from the nature or quality of the blood which distinguishes the cold-blooded from the warm-blooded vertebrata in the adult age. The same circumstance obtains with the other species of mammalia which lose their temperature and fall to the standard of the cold-blooded tribes. But in those (take as an instance the guinea-pig) which from the first day of its extra-uterine existence maintains its temperature nearly on a level with that of its parent when the air is temperate, the ductus arteriosus is closed immediately after birth.

This relation, continues our author, is preserved in the young mammalia in every modification in a peculiarly interesting manner. The young mammalia which are born with the eyes closed, at first present the phenomena of refrigeration nearly in the same degree during the two or three first days of their life; though they afterwards exhibit differences of great extent in this respect. Thus a young rabbit two days old had cooled down to 14° from 23° c, (to 58° from 74° F.) in the course of three hours fifty minutes, the air being at the time temperate; another three days old took seven hours twenty-five minutes to cool through a range of 18° c., when the process of refrigeration ceased. A third, of the age of five days only, lost 5° c. in temperature in the course of one hour fifty-five minutes, and maintained itself afterwards at this temperature. During the following days, smaller and smaller depressions of temperature were observed, till the eleventh day after birth, when the power of sustaining the temperature a little below that of the adult female parent seemed to be acquired permanently. When the modifications of internal structure are examined during this interval of time, we find that the ductus arteriosus has been contracting in the same proportion as the faculty of maintaining the temperature has been increasing, and that it is entirely closed at the epoch when the temperature becomes stationary, the external temperature being understood all the while as mild or pleasant. At the same period precisely too, the eyes are unsealed.

Among the young of birds, there are equally marked differences in the calorific function. Some lose heat rapidly when separated from the mother; others maintain their temperature to within a little of that of their species. Sparrows, for instance, which have been hatched but a short while, present a temperature from 4° to 5° c. lower than that of their parents when still contained in the nest, where they contribute to each other's warmth. But taken out of the nest and isolated, although the temperature be that of summer, they begin to cool with extreme rapidity. A young sparrow a few days old lost as many as 12° c. in the short space of one hour seven minutes, the air at the time marking 22° c. (72° F.). The same thing happens with swallows, sparrow-hawks, &c. But the law is not universal; it does not hold in reference to all the genera. There are several that have the power of sustaining their temperature in spring and summer at a degree but little below that of their parents. Birds, therefore, form two groups as regards the production of temperature, just as the mammalia do. The first cool down to the standard of cold-blooded animals; the second preserve their warmth when the air is mild or agreeable as it is in the spring and summer. It is interesting to inquire, if there is any external zoological character which marks the power or the incapacity to preserve heat. It is found in the presence or absence of feathers. Dr. Edwards asserts that though the absence of feathers marks and may contribute to the diminished faculty of maintaining a high temperature, it is not its main cause. The internal modification of organization which corresponds with this external sign is not so palpable as the patency of the ductus arteriosus in the young mammalia. But, in the first place, the nervous system is much less energetic in the unfeathered than in the feathered class; and, in the second place, the digestive powers are in an equal degree inferior in strength ; for they are not only unable to take food of themselves from muscular incapacity, but also from the lack of the requisite instinct, and, farther, from their digestive organs not being in a condition to elaborate food to any extent. It is on this last account that the parents supply their young with food which has suffered maceration in their own crops, or has even in their stomachs undergone a kind of incipient or partial solution ; or otherwise the parents have the instinct to select such articles as are easiest of digestion, and best fitted for the weakly state of the digestive organs of their progeny. We have already observed that a defect in the powers of digestion implies a corresponding imperfection in the blood.

The same principle applies to the first period in the existence of all animals. All have a temperature lower than that of their parents—all display inferiority of energy in their nervous system—all have less vigour of the digestive function and nutrition—all, in short, have the great functions of the body in a state of weakness, and the function of calorification, the result, in a great degree, of all, is weak too.

It follows that, if this be true, the prematurely born should have still less power of maintaining heat than those that are born at the full time. And so it would seem to be. Dr. Edwards made some observations on a case that presented itself to him. A child born at the seventh month, perfectly healthy, and which had come into the world with so little difficulty that the accoucheur could not be fetched in time to receive it, had been well clothed near a good fire when the temperature was taken at the axilla. This was found no higher than 32° c. (under 90° F.). Now we have seen that the mean of the temperature of ten children born at the full time was 34°, 75 c. (94°, 5 F.); the temperature in no case descending lower than 34° (94° F.), and ranging between this and 35°, 5 c. (96° F.)

Dr. Edwards makes some further remarks, the gist of which is that :—There are four states of the temperature from birth up to adolescence inclusive. In the first period the temperature is at the minimum ; in the second, it attains the adult degree : this might be entitled the period of the mean temperature ; in the third, the temperature exceeds that of the adult; finally, in the fourth, it sinks to the mean, that is, the temperature of the adult.

*Differences of Constitution in relation to the Production of Heat among Animals.*—It is obvious that animals may in a certain temperature display nearly the same temperature, or have a particular ratio of heat to each other, and yet when the surrounding temperature is altered, they may no longer exhibit the same, or maintain the ratio they formerly exhibited. And such is found to be the case ; indeed the preceding observations are themselves examples of the fact. But the subject is so eminently practical that it deserves to be investigated, at all events in the case of the young and the old. Dr. Edwards remarks :—

A young guinea-pig a month old, the temperature of whose body was high and steady, the temperature of the external air being mild, was exposed along with an adult to the same degree of diminished temperature—

the air was at 0° c. (32° F.). In the course of an hour the young creature had lost 9° c. in temperature, whilst the adult had only lost 2°, 5 c. This experiment, repeated several times with the same species of animal, always gave the same result. Young and adult birds of the same species, treated in a similar manner, showed the same diversity in their powers of resisting the effects of external cold, from which we may infer that the law is quite general. Several young magpies, for instance, whose temperature was stationary in a mild spring atmosphere, were placed with an adult in air cooled to + 4° c. After the lapse of twenty minutes, one of the young ones was found to have lost 14° of temperature. The others, examined at intervals, none of which exceeded one hour and ten minutes in length, had cooled from 14° to 16° c. The adult bird, on the contrary, similarly circumstanced, did not suffer a greater depression of temperature than 3° c. The loss of heat sustained by the young birds was so great as to be incompatible with life, if continued; that endured by the old one was trifling in amount and not inconsistent with health.

The difference between the young and the old was too great to be explained by mere difference of size, or of quantum of plumage, &c. Dr. Edwards goes on to say :—

A difference of constitution must go for a great deal; there are inherent diversities of constitution, favourable or the reverse, to the production of heat. The truth of this conclusion appears much more clearly if we continue to subject young birds to the same kind of experiment at successive epochs not so close to one another. The rapid progress they make in the power of evolving heat is, indeed, a very remarkable fact. A few days later, and they lose temperature in a much less considerable degree when exposed to cold under the same circumstances, although there was little or no apparent difference in the external appearance of the birds. And this is a new and convincing proof that the inequality in the disposition to lose heat obvious at different periods of life under exposure to a low external temperature, is principally owing to inherent inequality in the faculty of producing caloric.

These observations and the facts they establish are of the highest moment. They bear on the treatment of the young of our own race. What can be more preposterous than the scanty clothing which modern fashion allows to children? Whilst their mothers are muffled to the eyes in furs and to the toes in flannels, their unhappy brats are paraded abroad with their legs and bottoms exposed to every blast, and their necks left open for the very purpose of inviting scrofula. Yet Nature gives the mother a greater power of resisting cold, than she grants to the child, so barbarously frozen for the sake of vanity and mode.

*Influence of Seasons in the production of Animal Heat.*—Dr. Edwards remarks that the opinion, that the evaporation from the surface of the body in summer compensates the augmented temperature without, and that the cessation of that evaporation in winter makes up the deficiency of heat externally, must be taken with some reservation. For, he says, in examining those species of cold-blooded animals which from their structure are liable to lose more by evaporation than any other animal, we see that no such compensation takes place. Frogs, for example, the skin of which is so soft and permeable, and whose bodies besides are so succulent that they must be presumed in the most favourable circumstances to sustain loss by evaporation, ought to preserve the same temperature in winter and in summer if the low temperature in winter were compensated by the excess of evaporation in proportion as the heat of the season augments. But we know that the temperature of these creatures follows to a very great extent, that of the atmosphere, between 0° and 25° c. (32° and 77° F.), differing at no time from it by more than a degree or two. The phenomenon here is simple, by reason of the slight evolution of caloric by the frog, and leaves no doubt upon the mind. We must, therefore, have recourse to other conditions, in order to explain the slight difference that is observed in the summer and winter temperature of man and other warm-blooded animals.

He concludes that the cause is to be found in internal conditions of the body. In winter there is a *more active production* with a *greater loss;* in summer a *less production* with a *smaller loss* of heat. In this way is there compensation, and a perfect equilibrium maintained at all seasons. To render this relation more evident, it may be expressed in another manner; as, for example, in summer the body receives more heat from without, and produces less ; in winter it receives less and produces more. It follows, as an obvious deduction that, if the faculty of producing heat is less in summer the temperature of the body will not be maintained to the same point in the two seasons under sudden exposure to the same degree of cold. Dr. Edwards put this conclusion to the test of experiment.

" The apparatus employed consisted of earthen vessels plunged amidst a quantity of melting ice. Air thus cooled soon reaches the point of extreme humidity. The air being at zero c. (32° F.), the animal is introduced, placed upon a stage of gauze to prevent its coming in contact with the moist and rapidly conducting surface of the vessel. A cover, also piled over with ice, is then placed over the apparatus, but so arranged as still to permit the ready renovation of the air contained in the interior. Still farther to secure the purity of the included air, a solution of potash, which of course absorbed the carbonic acid produced with avidity, occupied the bottom of the vessel. In winter, in the month of February, the experiment was made at the same time, upon five adult sparrows, which were all included in the apparatus. At the end of an hour they were found one with another to have lost no more than 0°, 4 c., or less than half a degree ; some of them having suffered no depression of temperature whatsoever, others having lost as much as, but none more than, 1° c. The temperature of the whole then remained stationary to the end of the experiment, which was continued for three hours. In the month of July the same experiment was performed upon four full-grown or adult sparrows. The temperature of these birds at the end of an hour had undergone a depression, the mean term of which was 3°, 62, and the extremes 6°, 5 and 2° c. At the end of the third hour the mean term of the refrigeration suffered was 6°, the extremes being 12° and 3°, 5 c. It ought to have been stated that in the experiment in the winter month, the birds had been for some time kept in a warm room, so that the sudden transition was the same in both instances, in the winter as well as the summer experiment. The diversity in the constitution of these birds, consequently, with reference to the powers of producing heat, was an effect of the difference of the seasons. Each month the temperature of which differs in any degree from that of the month before or after it, has an obvious tendency to modify the temperament or constitution in the manner which has been indicated. In summer we may presume, nay we may be certain, that the differences obtain in degree according to the mean intensity of the heat proper to each. This is even to be proved by direct experiment. The month of August, as commonly happens, was not so hot as the month of July, and six sparrows treated in the same manner as those that were the subject of the July experiment already detailed, were not found to suffer refrigeration to the same extent. After the lapse of an hour the mean temperature of the six had sunk 1°, 62, and after three hours 4°, 87 c., from which it is obvious that with the successive declensions of the external temperature the faculty of engendering heat increases." 670.

Dr. Edwards compares the summer constitution with that of youth—the winter one with that of adult age—and the constitution of the natives of hot climates with that of the natives of temperate climates in summer. We pass over his observations on hybernation, having devoted so large a space to an article on the same subject by Dr. Marshall Hall.

*Of the System on which the External Temperature acts primarily and principally.*—Our sensations inform us that this is the nervous.

*Influence of Temperature on the Vitality of Cold-blooded Animals.*—If several cold-blooded animals, for example batrachia, have the lungs and heart excised, so that only the nervous system remains, and if, in this condition, they are plunged in water deprived of air, they will live in it different spaces of time according to the degree of its temperature, the extremes compatible with their existence being zero and 40 c. It is towards the inferior limit, zero, that they live the longest. Towards the upper limit they die almost immediately. Temperature, consequently presents in the scale of variations just mentioned very remarkable relations with the vitality of these animals. Towards the lower limit or that of melting ice, it is obviously most favourable to life; towards the upper limit, it is most inimical to life, extinguishing it almost immediately. Here it is impossible to mistake the system upon which the variety of temperature exerts its first and principal effects—the nervous system.

If respiration only be annihilated by plunging these creatures under water deprived of air, the temperature of which is caused to vary as above, they will be found to present the same phenomena according to the degree of the heat or cold, but in a more striking manner. Temperature in this case has the same kind of influence, but the effects are more manifest, from the circulation of the venous blood prolonging life at every degree short of the one at the upper limit of the scale, at which life is extinguished quite as suddenly as in the former instance.

If these experiments are made in Autumn the animals will be found to survive twice as long as they did in Summer, and if the experiments are repeated in Winter, the tenacity of life will be found to have increased in a very high degree.

*Influence of Temperature on the Vitality of Warm-blooded Animals.*—Dr. Edwards remarks:—

"Warm-blooded animals having in general a high temperature at all seasons of the year, they must be compared in this respect with cold-blooded animals in the height of summer. On the one hand, heat with certain limits tends to increase sensibility and motility; warm-blooded animals, therefore, with a few exceptions, which always present a high temperature, constantly exhibit also, with a few exceptions, a high degree of sensibility and motility. The same thing can only be said of the cold-blooded tribes during the continuance of the warm weather, on the other hand, again, high temperature tends to lessen the vitality proper to the nervous system, or the faculty of living without the agency of the ordinary stimuli. This is also the reason why, if respiration be interrupted among warm-blooded animals at all times, and among cold-blooded animals during the warmer seasons of the year, they all perish alike speedily, or nearly so. The difference in the time that elapses before life is extinct still depends on, or is in relation with, the difference of temperature. For in the hotter season of the year, cold-blooded animals never attain the temperature of the warm-blooded tribes, even in the most burning climates of the globe. Their nervous system will consequently have a higher degree of vitality in the sense already indicated; that is to say, they will not perish so promptly in summer under deprivation of air; but if they be immersed in water at the mean temperature of warm-blooded animals generally, which is about 40° c. (104° F.), they will die as suddenly—(at least this is the case with those of small size upon which the experiment has been made)—as the warm-blooded vertebrata when deprived of the contact of air." 674.

The effect of cold (short of a destructive quantum of it) on the vertebrata is to produce reaction. This may be only capillary, in the part, or it may extend to increased frequency of the respiratory and circulatory movements. Exercise quickens these movements materially and enables the animal to resist cold more effectually. By habit, as is well known, this power of reaction may be augmented, and low temperature borne with facility.

Our space is so limited that we must pass over a few pages, and content ourselves with the following quotation. It goes to confirm the preceding detail.

"Legallois, by the employment of various means for impeding respiration, or limiting the consumption of air, found that *refrigeration of animals is in the compound ratio of the difficulty experienced in breathing and of the quantity of oxygen consumed; so that when, in two experiments, the difficulty of breathing is the same, the greatest extent of cooling occurs in that in which the smallest quantity of oxygen is vitiated, and the contrary.* Now, the end of the process of respiration being to change the venous into arterial blood, this conclusion of Legallois confirms directly the one of the two principal conditions—THE STATE OF THE BLOOD, which we have laid down as influencing the production of heat among animals, and to the knowledge of which we had attained by induction.

The results of the direct experiments which we have still to quote also come powerfully in aid of our inferences concerning the other principal condition, which we have assumed from induction, influencing the production of animal heat: this is THE STATE OR ACTION OF THE NERVOUS SYSTEM.

Sir B. Brodie demonstrated by a series of the most ingeniously conceived and happily executed experiments, that when animals were decapitated and respiration was kept up by artificial means, so that the blood circulated as usual, and the process of change from the venous to the arterial state, went on uninterruptedly, the ordinary quantity of carbonic acid being eliminated, all the while, that, nevertheless, the temperature fell rapidly, even more rapidly than when no artificial respiration was maintained.

Dr. Chossat completed these researches upon the nervous system in its relations with the production of heat, by demonstrating in a series of experiments the following very important fact, viz. *that the depression of animal heat is constantly in relation with lesions of the nervous system, whether these lesions implicate the cerebro-spinal system, or the system of the great sympathetic.*" 683.

Here we must pause. We have been unable to turn to Müller. But as there are one or two questions connected with the production of animal heat untouched we may on a future occasion recur very briefly to them.

